# Supplementary material for: Picrotoxin Delineates Different Transport Configurations for Malate and γ Aminobutyric Acid through TaALMT1
Source: Biology (Basel). 2022 Aug 2;11(8):1162. doi: 10.3390/biology11081162 (PMC9405015; doi:10.3390/biology11081162)
Supplement: Supplementary file 1 [file biology-11-01162-s001.zip › biology-1783364-supplementary.pdf]

Figure S1

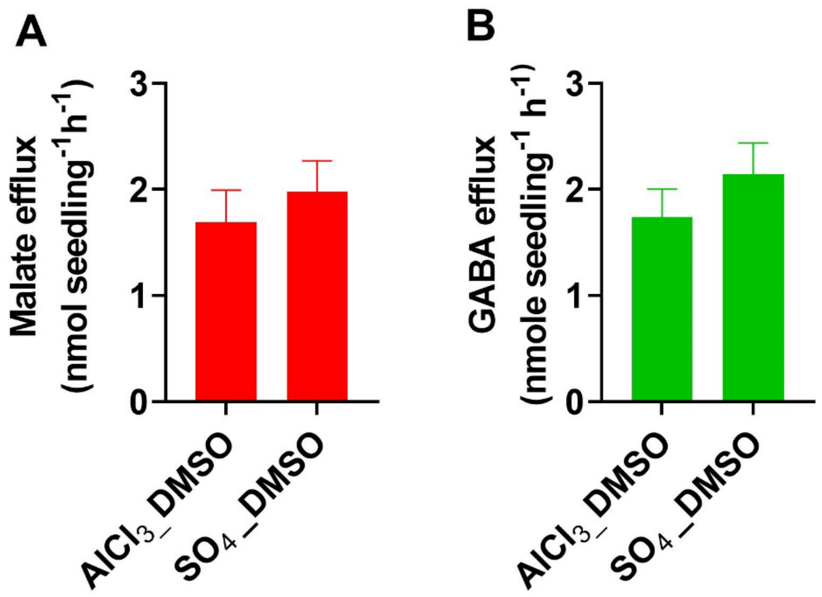

Figure S1. Dimethyl sulfoxide (DMSO) used to dissolve picrotoxin had no effect on malate efflux (**A**) or GABA efflux (**B**) from the wheat seedlings (ET8 NIL). This was tested by addition of DMSO to the 3-day old ET8 seedlings exposed to solutions containing 3 mM CaCl2 and 5 mM MES/BTP + AlCl3 (pH 4.5) or Na2SO4 (pH 7.5) and measuring the malate or GABA efflux after 22 h. Data shown (n = 5).

Figure S2

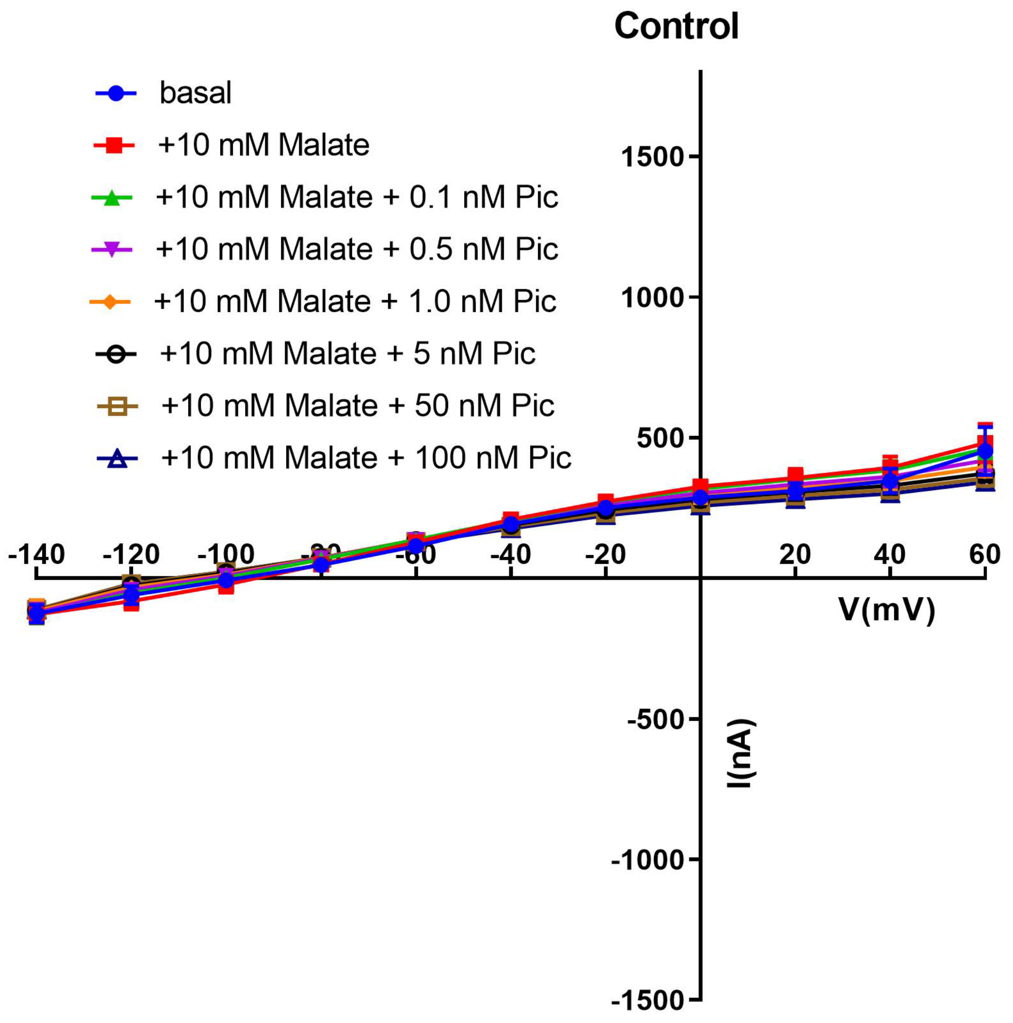

Figure S2. Picrotoxin had no effect on currents in control oocytes across a range of concentrations tested (0 – 100 nM) as shown in current-voltage (I/V) curves. Data presented here is from n = 5 Xenopus oocytes.
